# Commentary: Acute perturbation of *Pet1*-neuron activity in neonatal mice impairs cardiorespiratory homeostatic recovery

**DOI:** 10.3389/fphys.2019.00232

**Published:** 2019-03-18

**Authors:** Gregory D. Funk, Vivian Biancardi

**Affiliations:** Department of Physiology, Faculty of Medicine and Dentistry, Neuroscience and Mental Health Institute, Women and Children's Health Research Institute, University of Alberta, Edmonton, AB, Canada

**Keywords:** serotonin, autoresuscitation, SIDS, hypoxia, raphe

Sudden infant death syndrome (SIDS) is defined as “The sudden unexpected death of an infant <1 year of age, with onset of the fatal episode apparently occurring during sleep, that remains unexplained after a thorough investigation, including performance of a complete autopsy and review of the circumstances of death and the clinical history” (Krous et al., 2004). Despite its clinical definition as an unexplained death, epidemiology, pathology and sleep studies in humans combined with mechanistic studies of animal models have significantly advanced our understanding of this devastating syndrome (Li et al., 2018). This commentary highlights a report, and its potential relevance to SIDS, recently published in eLife by researchers at Harvard Medical School, Ryan Dosumu-Johnson and Susan M Dymecki, and the Geisel School of Medicine at Dartmouth, Andrea E Cocoran and Eugene Nattie (Dosumu-Johnson et al., 2018). Researchers describe a new mouse model and experiments that provide novel insight into the mechanisms of autoresuscitation, a vital reflex that is key for recovery from life-threatening apneas (periods when breathing stops) causally implicated in SIDS.

Parturition, the process of birth, is a complex event that transitions a fetus from the highly protected, climate-controlled, aquatic environment of the uterus to the harsh, external world. Successful transition requires precise coordination of multiple dynamic processes, perhaps the most dramatic of which occurs in the respiratory and cardiovascular systems; the placenta, responsible for fetal gas exchange, is suddenly abandoned and replaced with lungs that have just seen air and pulmonary circulation for the first time, and an immature respiratory pump and neural control system that, prior to birth, have only produced intermittent fetal breathing movements (Jansen and Chernick, 1991). Despite their immaturity, respiratory and cardiovascular systems must become fully functional at birth and their activities continuously coordinated by the developing brain for the newborn to survive. Failure, or improper development, of these neural control systems is believed to be a major contributor to SIDS. Almost 90% of SIDS cases occur between 2 and 4 months of age (Filiano and Kinney, 1994), suggesting that independent of the high risk associated with birth, there is a developmental period when cardiorespiratory networks are inherently unstable or that a delay or failure of a key development event creates a vulnerable period.

Identification of risk factors through epidemiological studies has greatly reduced the incidence of SIDS. Current rates [0.2–0.5 per 1,000 births in most countries (Goldstein et al., 2016)] have fallen to almost half of what they were in the early 90's, primarily due to the identification of prone sleeping as a major risk factor and the *Back to Sleep* public education campaign that encouraged caregivers to place their infants supine (on their back) to sleep. Despite this success, SIDS remains the leading cause of death in children <1 year of age in developed countries (Hauck et al., 2017).

Important mechanistic insight came from histological analyses of brain tissue from SIDS cases. Multiple brain abnormalities were identified in a host of brainstem modulatory systems, including catecholaminergic, cholinergic, neuropeptidergic, and serotonergic (5HT), suggesting that no single neurotransmitter system is at fault (Harper and Kinney, 2010). However, the most consistent deficits were alterations in the 5HT signaling system within the brainstem as well as the cerebellum, the two brain regions that have arguably received the greatest attention in the context of SIDS (Lawson and Thach, 1977; Paterson et al., 2006; Duncan et al., 2010; Harper and Kinney, 2010). Although rare, sleep studies of infants who ultimately died of SIDS have also been very insightful. During sleep, decreases in blood oxygen and increases in CO_2_ levels that develop during apnea typically cause arousal and restoration of normal breathing and heart rate. If this early defense mechanism fails, profound brain hypoxia develops triggering a final defense mechanism, autoresuscitation, which is an emergency set of reflexes that typically start with hyperpneic breaths, followed by hypoxic apnea and then gasping, which either increases brain oxygen sufficiently to restore normal breathing and heart rate, or it fails leading to a terminal apnea and death (Poets et al., 1999; Fewell et al., 2000; Sridhar et al., 2003; Fewell, 2005; Kinney and Thach, 2009; Li et al., 2018). Data from infants who later died of SIDS revealed that during sleep these babies often experienced multiple episodes of hypoxia, frequent gasping, tachycardia and bradycardia for days or hours before death; i.e., they successfully recovered (autoresuscitated) from multiple hypoxic apneas before succumbing to a terminal apnea when autoresuscitation failed (Poets et al., 1999; Sridhar et al., 2003).

Combined, these histological and sleep data suggest a role for 5HT signaling in autoresuscitation; i.e., deficiencies in 5HT signaling somehow make infants less able to autoresuscitate. Multiple animal models show a strong association between reduced levels of 5HT in the brain, impaired autoresuscitation, and reduced ability to survive repeated bouts of extreme hypoxia (Erickson and Sposato, 2009; Cummings et al., 2011; Chen et al., 2013; Yang and Cummings, 2013; Barrett et al., 2016). However, the limitation with these studies is that the 5HT manipulations were long term, starting in embryonic or postnatal life such that potential compensatory mechanisms confound interpretation. The work by Dosumu-Johnson et al. addresses this issue with a new mouse model that uses a chemogenetic approach (clozapine-N-oxide, CNO) to activate the synthetic inhibitory G protein-coupled receptor hM4Di) to acutely inhibit *Pet1*-derived (5HT) neurons on postnatal day 8 (~equivalent in mouse terms to the 2–4 month developmental window in human infants when SIDS rates are highest). With this model, researchers directly examined the effect of inhibiting *Pet1*-derived (5HT) neurons on the autoresuscitation reflex; i.e., the cardiorespiratory responses to a series of apneas (induced by inhalation of 97%N_2_/3%CO_2_).

Key findings include that following inhibition of *Pet1*-derived neurons, mice took fewer gasps during recovery from repeated apneas and were more likely to die (fail to autoresuscitate), providing direct evidence of a role for these neurons in autoresuscitation. Data also showed that, in contrast to control pups where respiratory and heart rate components of the autoresuscitation response followed very similar time courses (i.e., they were coupled), inhibition of *Pet1*-derived neurons had a much greater effect on the respiratory component of the autoresuscitation reflex than the heart rate component (i.e., they were uncoupled), indicating differential control. Sparse clinical data underlie a long-held suspicion that cardiorespiratory uncoupling is a factor in SIDS, but evidence of such uncoupling during autoresuscitation is very novel. Not only was the autoresuscitation response to repeated apneas compromised after inhibition of *Pet1*-derived neurons, the gasping reflex itself was compromised. The first gasp response following the initial apnea featured a smaller first gasp that took longer to occur; the time between gasps was also increased. Thus, *Pet1*-derived, 5HT neurons are important in the rapid activation of gasping and the generation of the gasping pattern. Very exciting is that these features of the initial gasp response (and others) were predictive of outcomes; i.e., whether the animal would survive the repeated apneic challenges.

In summary, the study by Dosumu-Johnson et al. provides new, direct evidence that activity in *Pet1*-derived (5HT) neurons plays a critical role in the autoresuscitation reflex, especially its respiratory (gasping) limb by promoting its rapid and full activation and proper coupling to the independently controlled cardiovascular limb of the reflex. While not direct evidence of a role in SIDS, these data provide a strong link between the serotonergic system, which consistently shows abnormal pathology in brain tissue from SIDS cases, and the “last-chance” autoresuscitation reflex that appears to fail in SIDS. These data, including evidence that specific cardiorespiratory response profiles are predictive of autoresuscitation failure, offer hope that strategies for autoresuscitation and tools to identify infants at risk for SIDS may be on the horizon.

## Author Contributions

GF and VB contributed to the writing and revision of this commentary.

### Conflict of Interest Statement

The authors declare that the research was conducted in the absence of any commercial or financial relationships that could be construed as a potential conflict of interest.

## References

[B1] Barrett K. T. Dosumu-Johnson R. T. Daubenspeck J. A. Brust R. D. Kreouzis V. Kim J. C.. (2016). Partial raphe dysfunction in neurotransmission is sufficient to increase mortality after anoxic exposures in mice at a critical period in postnatal development. J. Neurosci. 36, 3943–3953. 10.1523/JNEUROSCI.1796-15.2016 27053202 PMC4821907

[B2] Chen J. Magnusson J. Karsenty G. Cummings K. J. (2013). Time- and age-dependent effects of serotonin on gasping and autoresuscitation in neonatal mice. J. Appl. Physiol. (1985) 114, 1668–1676. 10.1152/japplphysiol.00003.2013 23558391

[B3] Cummings K. J. Commons K. G. Hewitt J. C. Daubenspeck J. A. Li A. Kinney H. C.. (2011). Failed heart rate recovery at a critical age in 5-HT-deficient mice exposed to episodic anoxia: implications for SIDS. J. Appl. Physiol. (1985) 111, 825–833. 10.1152/japplphysiol.00336.2011 21680874 PMC3174796

[B4] Dosumu-Johnson R. T. Cocoran A. E. Chang Y. Nattie E. Dymecki S. M. (2018). Acute perturbation of Pet1-neuron activity in neonatal mice impairs cardiorespiratory homeostatic recovery. Elife 7:e37857. 10.7554/eLife.37857 30350781 PMC6199134

[B5] Duncan J. R. Paterson D. S. Hoffman J. M. Mokler D. J. Borenstein N. S. Belliveau R. A.. (2010). Brainstem serotonergic deficiency in sudden infant death syndrome. JAMA 303, 430–437. 10.1001/jama.2010.45 20124538 PMC3242415

[B6] Erickson J. T. Sposato B. C. (2009). Autoresuscitation responses to hypoxia-induced apnea are delayed in newborn 5-HT-deficient Pet-1 homozygous mice. J. Appl. Physiol. (1985) 106, 1785–1792. 10.1152/japplphysiol.90729.2008 19213929

[B7] Fewell J. E. (2005). Protective responses of the newborn to hypoxia. Respir. Physiol. Neurobiol. 149, 243–255. 10.1016/j.resp.2005.05.006 15941675

[B8] Fewell J. E. Smith F. G. Ng V. K. Wong V. H. Wang Y. (2000). Postnatal age influences the ability of rats to autoresuscitate from hypoxic-induced apnea. Am. J. Physiol. Regul. Integr. Comp. Physiol. 279, R39–46. 10.1152/ajpregu.2000.279.1.R39 10896862

[B9] Filiano J. J. Kinney H. C. (1994). A perspective on neuropathologic findings in victims of the sudden infant death syndrome: the triple-risk model. Biol. Neonate 65, 194–197. 10.1159/000244052 8038282

[B10] Goldstein R. D. Trachtenberg F. L. Sens M. A. Harty B. J. Kinney H. C. (2016). Overall postneonatal mortality and rates of SIDS. Pediatrics. 137:e20152298. 10.1542/peds.2015-2298 26634772

[B11] Harper R. M. Kinney H. C. (2010). Potential mechanisms of failure in the sudden infant death syndrome. Curr. Pediatr. Rev. 6, 39–47. 10.2174/157339610791317214 22792083 PMC3392684

[B12] Hauck F. R. McEntire B. L. Raven L. K. Bates F. L. Lyus L. A. Willett A. M.. (2017). Research priorities in sudden unexpected infant death: an international consensus. Pediatrics 140:e20163514. 10.1542/peds.2016-3514 28751613

[B13] Jansen A. H. Chernick V. (1991). Fetal breathing and development of control of breathing. J. Appl. Physiol. (1985) 70, 1431–1446. 10.1152/jappl.1991.70.4.1431 2055820

[B14] Kinney H. C. Thach B. T. (2009). The sudden infant death syndrome. N. Engl. J. Med. 361, 795–805. 10.1056/NEJMra0803836 19692691 PMC3268262

[B15] Krous H. F. Beckwith J. B. Byard R. W. Rognum T. O. Bajanowski T. Corey T.. (2004). Sudden infant death syndrome and unclassified sudden infant deaths: a definitional and diagnostic approach. Pediatrics 114, 234–238. 10.1542/peds.114.1.234 15231934

[B16] Lawson E. E. Thach B. T. (1977). Respiratory patterns during progressive asphyxia in newborn rabbits. J. Appl. Physiol. Respir. Environ. Exerc. Physiol. 43, 468–474. 10.1152/jappl.1977.43.3.468 914718

[B17] Li A. Darnall R. A. Dymecki S. Leiter J. C. (2018). Animal models: illuminating the pathogenesis of sudden infant death syndrome, in SIDS. Sudden Infant and Early Childhood Death: The Past, the Present and the Future, ed. Duncan B.R.J. (Adelaide, SA: University of Adelaide; University of Adelaide Press), 759–828. 30035944

[B18] Paterson D. S. Trachtenberg F. L. Thompson E. G. Belliveau R. A. Beggs A. H. Darnall R.. (2006). Multiple serotonergic brainstem abnormalities in sudden infant death syndrome. JAMA 296, 2124–2132. 10.1001/jama.296.17.2124 17077377

[B19] Poets C. F. Meny R. G. Chobanian M. R. Bonofiglo R. E. (1999). Gasping and other cardiorespiratory patterns during sudden infant deaths. Pediatr. Res. 45, 350–354. 10.1203/00006450-199903000-00010 10088653

[B20] Sridhar R. Thach B. T. Kelly D. H. Henslee J. A. (2003). Characterization of successful and failed autoresuscitation in human infants, including those dying of SIDS. Pediatr. Pulmonol. 36, 113–122. 10.1002/ppul.10287 12833490

[B21] Yang H. T. Cummings K. J. (2013). Brain stem serotonin protects blood pressure in neonatal rats exposed to episodic anoxia. J. Appl. Physiol. (1985) 115, 1733–1741. 10.1152/japplphysiol.00970.2013 24136109

